# Supplementary material for: Daily rhythms in metabolic and locomotor behaviour of prematurely ageing PolgA mice
Source: FEBS Open Bio. 2024 Jul 28;14(10):1668–81. doi: 10.1002/2211-5463.13866 (PMC11452303; doi:10.1002/2211-5463.13866)
Supplement: Supplementary file 1 — Fig. S1. Daily cycle of locomotor activity of PolgA and WT mice. [file FEB4-14-1668-s002.pdf]

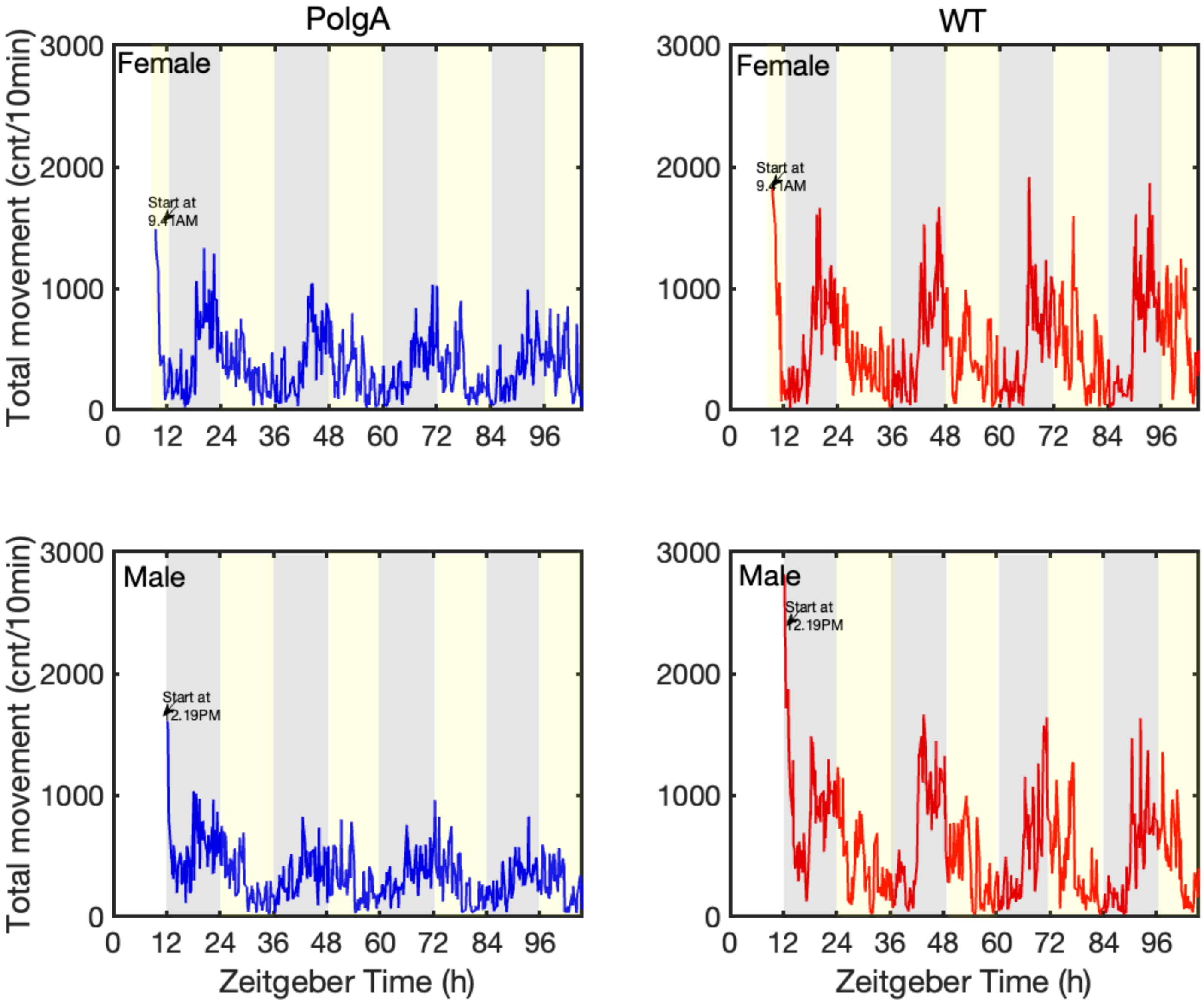

Supplementary Fig. S1: Daily cycle of locomotor activity of PolgA and WT mice. The data depict the total walking movements of PolgA (left panel) and WT (right panel) mice, and the measurements were recorded at 10-minute intervals over four consecutive days and nights. The data are presented as average values of locomotor activity (WT male (n=8), WT female (n=7), PolgA male (n=8), and PolgA female (n=8)).
