# Supplementary material for: Daily rhythms in metabolic and locomotor behaviour of prematurely ageing PolgA mice
Source: FEBS Open Bio. 2024 Jul 28;14(10):1668–81. doi: 10.1002/2211-5463.13866 (PMC11452303; doi:10.1002/2211-5463.13866)
Supplement: Supplementary file 2 — Fig. S2. Daily cycle of walking speed. [file FEB4-14-1668-s004.pdf]

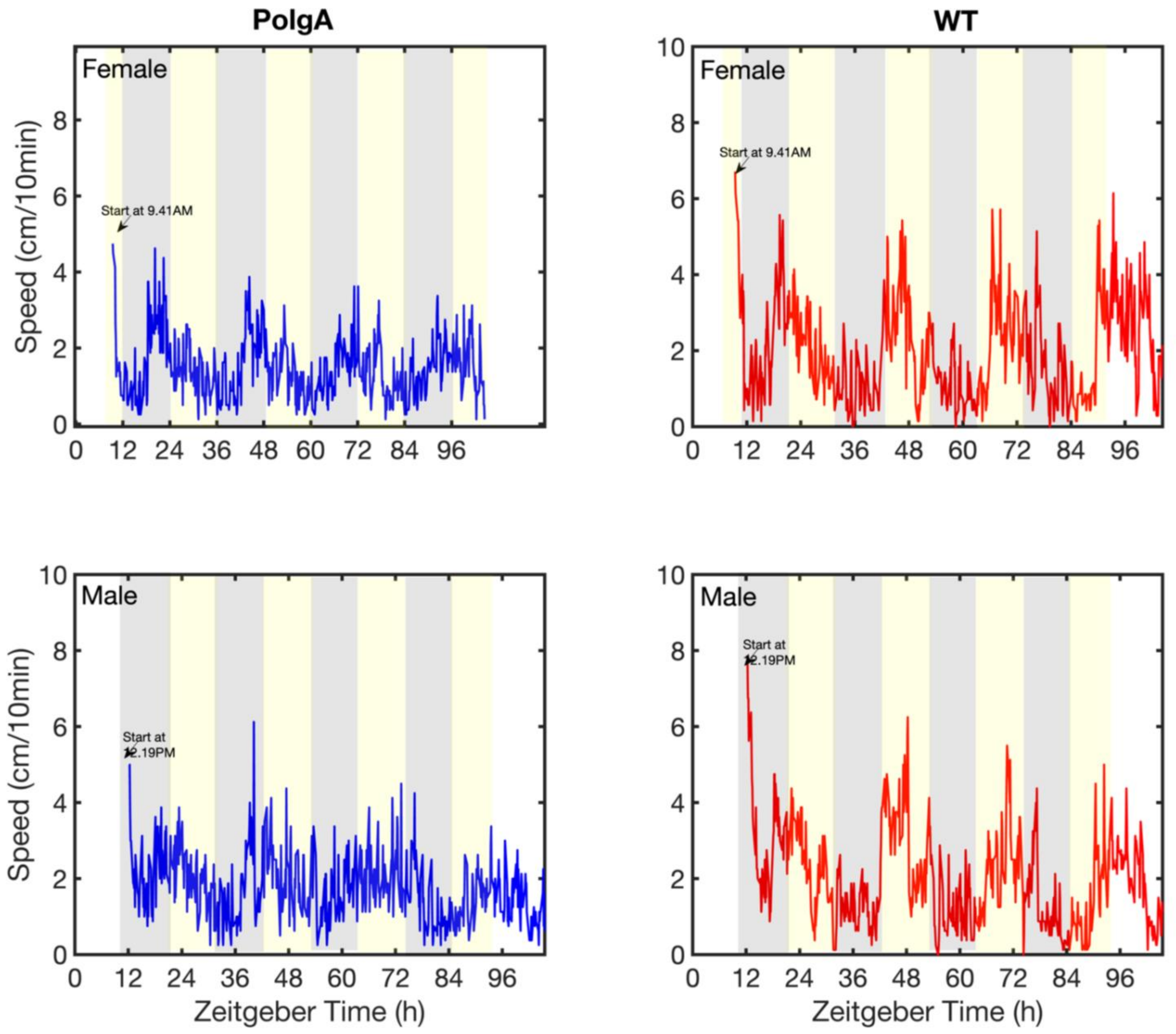

Supplementary Fig. S2: Daily cycle of walking speed.

The data illustrate the walking speed of PolgA mice (left panel) and WT mice (right panel) measured in the metabolic cage over four consecutive days and nights. The data are presented as average values of walking speed (WT male (n=8), WT female (n=7), PolgA male (n=8), and PolgA female (n=8)).
